# Association of niacin intake with constipation in adult: result from the National Health and Nutrition Examination

**DOI:** 10.1186/s40001-023-01362-6

**Published:** 2023-09-26

**Authors:** Xiao Huang, Liwen Zhao, Zhenyang Li, Xiaodong Gu, Mingzhe Li, Jianbin Xiang

**Affiliations:** 1 Department of Gastrointestinal Surgery, Huashan Hospital, 12 Middle Urumqi Road, Shanghai, 200040 China; 2 Clinical Medicine, Medical College of Wuhan University of Science and Technology, Wuhan, Hubei China; 3 Department of Anatomy, Medical College of Wuhan University of Science and Technology, Wuhan, Hubei China

**Keywords:** Niacin, Constipation, National Health and Nutrition Examination Survey

## Abstract

**Background:**

Although dietary intake is believed to be associated with constipation, there is currently a lack of research exploring the relationship between niacin intake and constipation. Therefore, the aim of this study is to investigate the association between niacin intake in adults and constipation using data from the National Health and Nutrition Examination Survey (NHANES).

**Methods:**

This study included 5170 participants (aged ≥ 20 years) from the NHANES survey conducted between 2009 and 2010. Participants who reported experiencing constipation “always”, “most of the time”, or “sometimes” in the past 12 months were defined as constipation cases. The daily niacin intake was obtained from dietary recall and dietary supplement recalls of the patients. Weighted multivariate logistic regression analysis, restricted cubic spline regression, subgroup analysis, and interaction analysis were used to assess the correlation between niacin intake and constipation.

**Results:**

After adjustment for covariates, the multivariate logistic regression model showed that low niacin intake was associated with a higher risk of constipation (Model 1: OR: 0.917, 95% CI 0.854–0.985, *P* = 0.023; Model 2: OR: 0.871, 95% CI 0.794–0.955, *P* = 0.01). After dividing niacin intake into four groups, a daily intake of 0–18 mg niacin was associated with a higher risk of constipation (Model 1: OR: 1.059, 95% CI 1.012–1.106, *P* = 0.019; Model 2: OR: 1.073, 95% CI 1.025–1.123, *P* = 0.013). The restricted cubic spline regression analysis also showed a non-linear relationship between niacin intake and the risk of constipation.

**Conclusion:**

The findings of this study suggested that daily intake of 0–18 mg of niacin was associated with a higher risk of constipation compared to a daily intake of 18–27 mg of niacin.

## Background

Constipation is a common chronic gastrointestinal disease characterized by symptoms such as difficulty in defecation, reduced frequency of defecation, and incomplete evacuation after defecation, which can significantly affect patients' quality of life and psychological health [1]. Due to the complex etiology of constipation, the American Gastroenterological Association has classified constipation patients into three categories: normal transit constipation, slow transit constipation, and pelvic floor dysfunction or defecatory disorders, in order to better guide treatment [2]. Various risk factors related to constipation have been extensively studied, including selenium intake [3], energy intake [4], and liquid intake [5]. However, Werth [6] found that the existing evidences on most constipation risk factors are contradictory or insufficient, thus large-scale studies including a wide range of factors in a community environment are necessary to further understand the risk factors of constipation.

Niacin is an organic water-soluble compound primarily metabolized in the liver, with its main metabolites being uric acid and nicotinamide. Niacin may play a role in various diseases. By affecting immune cells and vascular endothelial cells through its receptor, niacin exerts its anti-atherosclerotic effects [7]. Even in patients with metabolic syndrome, the cardiovascular benefits of combined therapy with niacin and simvastatin outweigh the adverse effects on blood glucose [8]. Retrospective studies have also indicated a negative correlation between niacin intake and the incidence of pancreatic cancer [9]. Moreover, niacin and its derivatives have been found to play important roles in regulating inflammatory responses and maintaining intestinal health [10, 11]. However, there is currently no study evaluating the effect of niacin intake on constipation.

Therefore, this study aims to investigate the relationship between niacin intake and constipation by utilizing the National Health and Nutrition Examination Survey (NHANES) database, while adjusting for confounding factors such as age, gender, and others dietary intake.

## Materials and methods

### Study subjects

The NHANES is a population-based cross-sectional survey that includes a series of cross-sectional data since 1998, designed to evaluate the health and nutritional status of adults and children in the United States. The survey utilizes a complex, stratified, multistage, probability cluster design to ensure that its data accurately reflect the health and nutritional status of the U.S. population. Detailed information about NHANES' continuous survey design can be found at http://www.cdc.gov/nchs/nhanes/index.htm. Written informed consent was obtained from all participants, and NHANES has been approved by the Ethics Review Board of the National Center for Health Statistics.

**Fig. 1:**
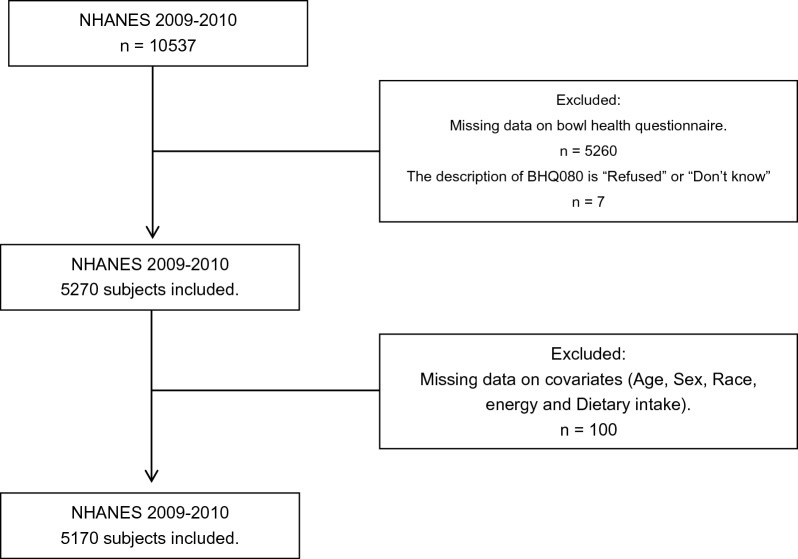
Flowchart of selection process

Participants aged ≥ 20 years who participated in NHANES (2009–2010) were included in this study, with a total of 10,537 participants. We excluded patients with missing data in bowel health questionnaire (*n* = 5267), and then individuals with missing information on confounding factors (age, sex, race, energy, and dietary intake) were also excluded from the study population (*n* = 100). Therefore, a total of 5170 patients were used for further analysis (2615 female and 2555 men) (Fig. 1).

### Definition of constipation

The Bowel Health Questionnaire from 2009–2010 is frequently used to identify research subjects with chronic constipation [3, 4, 12]. Participants were asked the question: “During the past 12 months, how often have you been constipated?” Patients who answered “always”, “most of the time”, or “sometimes” were considered to have constipation, while those who answered “rarely” or “never” were considered to have normal bowel movements.

### Niacin intake

The dietary intake data are obtained by recording the types and amounts of food and beverages consumed in the 24 h prior to the interview, and estimating the intake of energy, nutrients, and other food components in these foods and beverages. Participants undergo two 24-h dietary recall interviews, the first conducted at the Mobile Examination Center (MEC) and the second collected via telephone 3 to 10 days later. When both 24-h recalls are completed by a participant, we use the mean intake of niacin. If a participant responded to only the first interview, we use only the first 24-h intake of niacin. Similarly, we also use data from two 24-h dietary supplement recalls to assess the intake of niacin from dietary supplements consumed by participants and calculate total niacin intake. We then categorize niacin intake by quartiles: Q1 (0–18 mg), Q2 (18–27 mg), Q3 (27–39 mg), Q4 (> 39 mg).

### Covariates

We considered multiple factors as covariates between constipation and niacin intake, including age, gender, race, marital status, body mass index (BMI), alcohol consumption, smoking, and the intake of other dietary elements. Age was divided into three groups (20–45 years old, 45–65 years old, and 65 + years old); gender was divided into two groups (male and female); race was divided into non-Hispanic white, Mexican American, non-Hispanic black, other Latin American, and other; marital status was divided into widowed/divorced/separated, never married, married/living with partner; BMI was divided into four groups [underweight (< 18.5), normal (18.5 to < 25), overweight (25 to < 30), obese (30 +)]; alcohol consumption was divided into four groups (non-drinker, 1–5 drinks/month, 5–10 drinks/month, 10 + drinks/month); smoking status was categorized as never smoker, former smoker, and current smoker based on whether they had smoked 100 cigarettes in the past and current smoking status. Other dietary elements were presented as continuous variables, and if an element was present in both the dietary questionnaire and dietary supplement questionnaire, the total intake was obtained by merging the two data.

### Statistical analyses

Due to the non-normal distribution of niacin, log10 transformation was used before the data analysis. Continuous variables were described using median and interquartile range, while categorical variables were described using count and percentage. Wilcoxon rank-sum test was used to compare continuous variables between different groups, and Chi-squared test with Rao and Scott’s second-order correction was used to compare the categorical variables. Based on continuous and categorical forms of niacin, we constructed three logistic regression models to calculate the odds ratio (OR) and 95% confidence interval (CI) of niacin intake versus constipation: crude model without adjustment for confounding factors; model 1 adjusted for age, gender, and race; model 2 adjusted for energy, protein, carbohydrate, total sugars, total fat, total saturated fatty acids, total monounsaturated fatty acids, phosphorus, sodium, and potassium. Moreover, we employed additional analytical methods including restricted cubic splines to investigate the non-linear association between niacin intake and constipation. Furthermore, interaction analyses were conducted based on covariates such as age, gender, race, BMI, marital status, alcohol consumption, and smoking status, in order to examine their relationship with stratification variables.

Considering the complex sampling methods, we used corresponding sampling weights to obtain nationally representative estimates. All statistical analyses were performed in R version 4.2.2, and *P* < 0.05 was considered statistically significant.

## Result

### The characteristics of study participants

Overall, a total of 5170 patients were included in the study, including 1270 (22%) constipation patients and 3900 (78%) individuals with normal bowel movements. The distribution of weighted characteristics between the two groups is shown in Tables 1 and 2. For baseline data, there were significant differences in gender (*P* < 0.001), race (*P* = 0.009), marital status (*P* = 0.015), and alcohol consumption (*P* < 0.001) between constipation and normal bowel movement patients. Although constipation symptoms were more prevalent in the 65 + age group, there was no significant difference compared to the normal bowel movements group (18% vs. 15%, *P* = 0.077). The distribution of smokers was not significantly different between the two groups (*P* = 0.5) (Table 1). In terms of dietary intake, compared to patients with normal bowel movement, constipation patients consumed less niacin, energy, protein, carbohydrate, total sugars, total fat, cholesterol, moisture, total saturated fatty acids, total monounsaturated fatty acids, total polyunsaturated fatty acids, folic acid, vitamin E, phosphorus, sodium, and potassium. Surprisingly, there was no significant difference in fiber intake between the two groups (Table 2). Table 3 shows the univariate logistic regression results of covariates and constipation. Advanced age, female sex, Mexican American, Other Hispanic, and widowed/divorced/separated are risk factors for constipation. Interestingly, alcohol consumption was a protective factor for constipation. In terms of dietary, intake of more energy, protein, carbohydrates, total sugars, total fat, moisture, total saturated fatty acids, total monounsaturated fatty acids, vitamin E, phosphorus, sodium, and potassium are also protective against constipation.

**Table 1:** The baseline characteristics of constipation and normal participant

Characteristic	Normal, *N* = 3900 (78%)^a^	Constipation, *N* = 1270 (22%)^a^	*P*-value^b^
Age			0.077
20–45 years	75,882,410 (49%)	19,870,882 (44%)	
46–65 years	55,147,795 (36%)	16,854,954 (38%)	
65 + years	23,707,593 (15%)	7,994,405 (18%)	
Sex			< 0.001
Male	83,596,622 (54%)	14,251,719 (32%)	
Female	71,141,176 (46%)	30,468,521 (68%)	
Race			0.009
Non-Hispanic White	109,726,900 (71%)	29,105,702 (65%)	
Mexican American	12,048,999 (7.8%)	4,623,380 (10%)	
Non-Hispanic Black	16,599,399 (11%)	5,497,275 (12%)	
Other Hispanic	7,376,470 (4.8%)	2,863,968 (6.4%)	
Other	8,986,030 (5.8%)	2,629,915 (5.9%)	
Marital status			0.015
Widowed/divorced/separated	27,330,217 (18%)	9,046,101 (20%)	
Never married	30,288,229 (20%)	6,273,186 (14%)	
Married/living with partner	96,985,514 (63%)	29,400,953 (66%)	
BMI			0.6
Underweight (< 18.5)	2,775,474 (1.8%)	527,099 (1.2%)	
Normal (18.5 to < 25)	43,501,329 (28%)	12,905,615 (29%)	
Overweight (25 to < 30)	51,690,803 (33%)	14,732,326 (33%)	
Obese (30 or greater)	56,770,192 (37%)	16,555,200 (37%)	
Alcohol			< 0.001
Non-drinker	30,105,255 (19%)	12,504,728 (28%)	
1–5 drinks/month	73,992,095 (48%)	22,193,893 (50%)	
5–10 drinks/month	16,646,996 (11%)	3,516,618 (7.9%)	
10 + drinks/month	33,993,453 (22%)	6,505,001 (15%)	
Smoke			0.5
Never smoker	85,728,507 (55%)	23,644,148 (53%)	
Former smoker	38,726,830 (25%)	11,076,679 (25%)	
Current smoker	30,282,461 (20%)	9,999,413 (22%)	

^a^*n* (%)

^b^Chi-squared test with Rao and Scott's second-order correction

**Table 2:** Daily intake of constipated subjects and normal participant

Characteristic	Normal, *N* = 3907 (78%)^a^	Constipation, *N* = 1270 (22%)^a^	*P*-value^b^
Niacin			< 0.001
Q2 (18–27)	40,507,351 (26%)	11,510,966 (26%)	
Q1 (0–18)	28,026,902 (18%)	12,353,301 (28%)	
Q3 (27–39)	40,069,460 (26%)	10,367,768 (23%)	
Q4 (> 39)	46,134,085 (30%)	10,488,205 (23%)	
Energy	2024 (1548, 2608)	1815 (1437, 2305)	< 0.001
Protein	79 (60, 103)	71 (55, 91)	< 0.001
Carbohydrate	245 (187, 311)	227 (176, 291)	0.006
Total sugars	104 (68, 148)	98 (68, 136)	0.041
Fiber	16 (11, 22)	15 (11, 22)	0.3
Total fat	74 (53, 101)	66 (48, 88)	0.002
Cholesterol	240 (155, 368)	211 (142, 345)	0.001
Moisture	3950 (2704, 5752)	3649 (2602, 5554)	0.014
Total saturated fatty acids	24 (16, 34)	21 (15, 30)	0.002
Total monounsaturated fatty acids	26 (19, 36)	23 (17, 32)	0.001
Total polyunsaturated fatty acids	16 (11, 23)	15 (10, 21)	0.010
Folic acid	224 (119, 496)	192 (94, 469)	0.015
Vitamin E	6.9 (4.7, 9.9)	6.3 (4.3, 9.3)	0.022
Phosphorus	1346 (1038, 1759)	1238 (954, 1585)	0.001
Sodium	3406 (2566, 4426)	3072 (2333, 3925)	< 0.001
Potassium	2703 (2065, 3445)	2524 (1885, 3247)	0.003
Caffeine	128 (42, 251)	102 (32, 214)	0.005

^a^*n* (%); median (IQR)

^b^Chi-squared test with Rao and Scott’s second-order correction; Wilcoxon rank-sum test for complex survey samples

**Table 3:** Univariate logistic regression of the association between all variables and constipation

Characteristic	Constipation, normal	*P*-value
Age		
20–45 years	–	–
45–65 years	1.027 (0.991, 1.064)	0.13
65 + years	**1.046 (1.006, 1.086)**	**0.025**
Sex		
Male	–	–
Female	**1.167 (1.129, 1.206)**	**< 0.001**
Race		
Non-Hispanic White	–	–
Mexican American	**1.07 (1.029, 1.113)**	**0.003**
Non-Hispanic Black	1.04 (0.997, 1.085)	0.067
Other Hispanic	**1.073 (1.017, 1.131)**	**0.015**
Other	1.017 (0.946, 1.093)	0.6
Marital status		
Widowed/divorced/separated	–	–
Never married	**0.926 (0.884, 0.969)**	**0.003**
Married/living with partner	0.984 (0.942, 1.028)	0.4
BMI		
Underweight (< 18.5)	–	–
Normal (18.5 to < 25)	1.072 (0.998, 1.15)	0.054
Overweight (25 to < 30)	1.064 (0.979, 1.156)	0.13
Obese (30 +)	1.068 (0.98, 1.165)	0.12
Alcohol		
Non-drinker	–	–
1–5 drinks/month	**0.939 (0.901, 0.979)**	**0.006**
5–10 drinks/month	**0.888 (0.819, 0.962)**	**0.007**
10 + drinks/month	**0.876 (0.836, 0.917)**	**< 0.001**
Smoke		
Never smoker	–	–
Former smoker	1.006 (0.954, 1.06)	0.8
Current smoker	1.033 (0.98, 1.088)	0.2
Energy	**0.9999552 (0.9999344, 0.9999761)**	**< 0.001**
Protein	**0.9989638 (0.9984574, 0.9994705)**	**< 0.001**
Carbohydrate	**0.9997679 (0.999618,0.9999179)**	**0.005**
Total sugars	**0.9997655 (0.9995765, 0.9999546)**	**0.018**
Fiber	0.999071 (0.9971961, 1.000949)	0.3
Total fat	**0.9992993 (0.998814, 0.9997849)**	**0.008**
Cholesterol	0.9998913 (0.9997802, 1.000002)	0.055
Moisture	**0.9999931 (0.9999879, 0.9999983)**	**0.013**
Total saturated fatty acids	**0.9980179 (0.9968362, 0.999201)**	**0.003**
Total monounsaturated fatty acids	**0.9979532 (0.9965832, 0.9993251)**	**0.006**
Total polyunsaturated fatty acids	0.9987412 (0.996831, 1.000655)	0.2
Folic acid	0.9999492 (0.9998941, 1.000004)	0.068
Vitamin E	**0.9969062 (0.9944602, 0.99936)**	**0.017**
Phosphorus	**0.9999426 (0.9999109, 0.9999743)**	**0.002**
Sodium	**0.9999782 (0.9999636, 0.9999928)**	**0.006**
Potassium	**0.99998 (0.9999675, 0.9999925)**	**0.004**
Caffeine	0.9999387 (0.9998738, 1.000004)	0.062

Bold values indicates significance level of p < 0.05

OR are displayed with their 95% confidence intervals and *P*-value

### Associations between niacin intake and risk of constipation

**Fig. 2:**
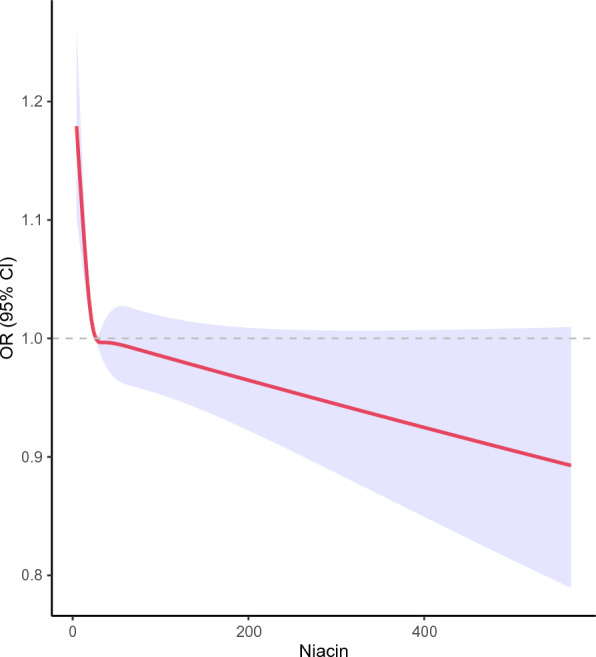
Association between niacin intake and constipation. Adjustment factors included energy, protein, carbohydrate, total sugars, total fat, total saturated fatty acids, total monounsaturated fatty acids, phosphorus, sodium, potassium.

The relationship between niacin intake and constipation is shown in Table 4. In the unadjusted model, there was a significant negative correlation between log-transformed niacin intake and constipation (OR: 0.849, 95%CI 0.793–0.909, *P* < 0.001). This significant association remained after adjusting for covariates (Model 1: OR: 0.917, 95%CI 0.854–0.985, *P* = 0.023; Model 2: OR: 0.871, 95%CI 0.794–0.955, *P* = 0.01). When niacin was converted into a four-category variable, participants in the Q1 group (0–18 mg) had a significantly higher risk of constipation compared to those in the Q2 group (18–27 mg) (crude model: OR: 1.088, 95%CI 1.046–1.132, *P* < 0.001; Model 1: OR: 1.059, 95%CI 1.012–1.106, *P* = 0.019; Model 2: OR: 1.073, 95%CI 1.025–1.123, *P* = 0.013). However, there was no significant association between niacin intake and the risk of chronic constipation in the Q3 and Q4 groups. Restricted cubic spline models showed a non-linear relationship between niacin intake and the OR of chronic constipation (Fig. 2). Once the intake of niacin exceeded 27 mg, the protective effect of niacin became noticeably attenuated.

**Table 4:** Logistic regression of the association between niacin intake and constipation

Characteristic	Constipation, normal	*P*-value
Crude model		
Continuous [log_10_(niacin)]	**0.849 (0.793, 0.909)**	**< 0.001**
Q2 (18–27 mg)	–	–
Q1 (0–18 mg)	**1.088 (1.046, 1.132)**	**< 0.001**
Q3 (27–39 mg)	0.984 (0.936, 1.036)	0.5
Q4 (> 39 mg)	0.965 (0.910, 1.022)	0.2
Model 1		
Continuous [log_10_(niacin)]	**0.917 (0.854, 0.985)**	**0.023**
Q2 (18–27 mg)	–	–
Q1 (0–18 mg)	**1.059 (1.012, 1.106)**	**0.019**
Q3 (27–39 mg)	0.998 (0.949, 1.106)	> 0.9
Q4 (> 39 mg)	0.997 (0.939, 1.058)	0.9
Model 2		
Continuous [log_10_(niacin)]	**0.871 (0.794, 0.955)**	**0.01**
Q2 (18–27 mg)	–	–
Q1 (0–18 mg)	**1.073 (1.025, 1.123)**	**0.013**
Q3 (27–39 mg)	0.990 (0.928, 1.056)	0.7
Q4 (> 39 mg)	0.978 (0.904, 1.058)	0.5

Bold values indicates significance level of p < 0.05

OR are displayed with their 95% confidence intervals and *P*-value. Model 1 was adjusted for none; Model 2 adjusted for age, sex and race; Model 3 adjusted for energy, protein, carbohydrate, total sugars, total fat, total saturated fatty acids, total monounsaturated fatty acids, phosphorus, sodium, potassium

### Subgroup analyses

**Fig. 3:**
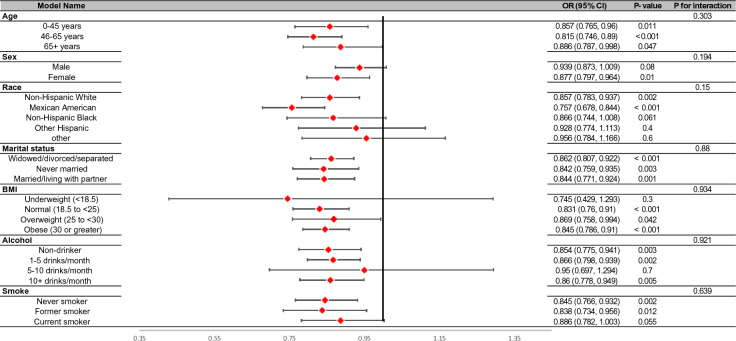
Stratified analyses by potential modifiers of the association between niacin intake and constipation

Participants were divided into different subgroups according to age, gender, race, marital status, BMI, alcohol consumption, and smoking status to evaluate the relationship between log10 (niacin) intake and constipation in different subgroups (Fig. 3). However, these variables did not significantly affect the relationship between niacin intake and chronic constipation (*P* > 0.05).

## Discussion

This research examined the correlation between daily niacin intake and the incidence of constipation in adults. Constipation was defined in this study based on patients’ recollections of episodes experienced within the past year. The findings indicated a negative correlation between niacin intake and the incidence of constipation. An intake of 0–18 mg/day of niacin was identified as a risk factor for constipation, whereas intakes of 27–39 mg/day and > 39 mg/day did not significantly decrease the risk of constipation, as compared to an intake of 18–27 mg/day. It should be noted that adverse effects, such as vasodilation, may occur when niacin intake exceeds 50 mg/day [13]. Therefore, it is advisable to recommend the appropriate supplementation of niacin for constipation patients in clinical settings.

Given that the NHANES bowel questionnaire BHQ060 was built upon the well-established Bristol stool form scale [14], previous studies often relied upon this criterion for defining constipation [3, 4, 12]. However, individuals' perception of bowel movements and the actual form of feces might not align perfectly. Moreover, there was an increasing emphasis on patients' self-perceived symptoms. Thus, this study defined constipation based on self-reported symptoms. Prior research had demonstrated that the incidence of constipation varies depending on the definition used. When compared to other diagnostic criteria, self-reported symptoms lead to a higher incidence rate [15]. In this study, the incidence of constipation was found to be 22%, notably surpassing the rates obtained using the Rome I/II/III diagnostic criteria (10.1–15.3%). Additionally, the distribution of incidence between different genders appeared similar [16].

In previous studies of pellagra, researchers found that patients with niacin deficiency exhibited gastrointestinal symptoms, but the underlying mechanism remains unclear [17]. Singh et al. [18] discovered that niacin bound to GPR109A to promote the production of interleukin-10 (IL-10) in mouse macrophages and dendritic cells, further inducing T cell differentiation. Meanwhile, the expression of IL-18 was also regulated by GPR109A. Therefore, in mice with niacin deficiency, the repair function of intestinal mucosal barrier was impaired, making them more susceptible to intestinal inflammation. Maintaining the stability of the intestinal microecology was also considered an important way in which GPR109A promoted intestinal mucosal barrier repair [19], and research had shown that the use of antibiotics to induce gut microbiota depletion inhibited the expression of IL-23 and ILC3 in GPR109A knockout mice, reducing intestinal inflammation [20]. Coenzymes NAD + and NADP + , metabolites of niacin, played an important role in metabolism and energy release. Research found that raising the level of NAD + enhanced oxidative metabolism, providing protection against high-fat diet-induced metabolic abnormalities [21]. Similarly, Gomes et al. [22] found that increasing NAD + levels in elderly mice can restore mitochondrial function to the level of young mice. SIRT1 was a NAD + -dependent histone deacetylase that promoted the phosphorylation of S6K1, thereby increasing the number of intestinal stem cells [23]. Additionally, SIRT1 could prevent intestinal inflammation by regulating the gut microbiota [11]. Given the close relationship between niacin and intestinal function, it was necessary to evaluate the association between niacin intake and constipation. In addition, the research indicated that female participants had a higher risk of constipation, which was consistent with previous research results [4, 12]. However, further exploration is needed to elucidate the mechanism underlying gender differences in the risk of constipation. In further analysis, there was no interaction between niacin intake and gender.

This study has several limitations. Firstly, the NHANES database contains cross-sectional data, so this study cannot establish a causal relationship between constipation and niacin intake. Secondly, the use of recalled data may introduce potential recall bias. Thirdly, the NHANES database did not provide more detailed data, and there may be other potential confounding factors related to constipation that were not included in the study. Finally, the estimation of dietary intake elements through questionnaire-based calculations represents a coarse assessment. Furthermore, a subset of patients lacking follow-up information was excluded from the analysis, both of which inevitably contribute to confounding factors. On the other hand, this study has several strengths: (1) it includes a large nationally representative sample, which makes the results more convincing; (2) unlike other studies on constipation that used the NHANES database [3, 4, 12], this research used a definition of constipation derived from participants' self-reported symptoms, thereby placing a heightened emphasis on individuals who manifest constipation-related complaints despite yielding negative results on the Bristol Stool Scale assessment. (3) To the best of our knowledge, there is currently a lack of research on the relationship between daily niacin intake and constipation, so this study fills this gap.

## Conclusions

In summary, our study suggests a potential association between low niacin intake and increased risk of constipation. Furthermore, we found no significant association between niacin intake in the range of 27–39 mg/day and > 39 mg/day and higher risk of constipation compared to the reference group (18–27 mg/day). Thus, optimal niacin intake should be considered for a healthy lifestyle. Further research is needed to confirm the potential benefits of appropriate niacin intake in improving constipation and to elucidate the underlying mechanisms.

## Data Availability

The data of this study can be available by contacting the corresponding author.

## Abbreviations

NHANES: National Health and Nutrition Examination Survey
MEC: Mobile Examination Center
BMI: Body mass index
OR: Odds ratio
CI: Confidence interval
IL-10: Interleukin-10
